# *Withania somnifera (L.) Dunal* as Add-On Therapy for COPD Patients: A Randomized, Placebo-Controlled, Double-Blind Study

**DOI:** 10.3389/fphar.2022.901710

**Published:** 2022-06-16

**Authors:** Priyam Singh, Khushtar Anwar Salman, Mohammad Shameem, Mohd Sharib Warsi

**Affiliations:** ^1^ Department of Biochemistry, Jawaharlal Nehru Medical College, Aligarh Muslim University, Aligarh, India; ^2^ Department of Tuberculosis and Respiratory Diseases, Jawaharlal Nehru Medical College, Aligarh Muslim University, Aligarh, India

**Keywords:** complementary medicine, COPD, FEV1% predicted, interleukin-6, *in silico*, myeloperoxidase, neutrophil, *Withania somnifera*

## Abstract

**Background:** The current gold-standard therapies for chronic obstructive pulmonary disease (COPD) lack disease-modifying potential and exert adverse side effects. Moreover, COPD patients are at a higher risk of severe outcomes if they get infected by severe acute respiratory syndrome coronavirus 2 (SARS-CoV-2) virus, the cause of the current epidemic. This is the first study to document clinical research on an adaptogenic and steroidal activity–containing herb as a complementary medicine for COPD treatment.

**Objective:** We aimed to evaluate the efficacy of *Withania somnifera* (*L.*) *Dunal* [*Solanaceae*] (WS) as an add-on therapy for COPD patients.

**Methods:** A randomized, placebo-controlled, and double-blind clinical study was conducted. A total of 150 patients were randomly assigned to three groups: control, placebo, and WS group. In addition to conventional medicines, WS root capsules or starch capsules were given twice a day to the WS group and the placebo group, respectively. Their lung functioning, quality of life, exercise tolerance, systemic oxidative stress (OS), and systemic inflammation were assessed before and after 12 weeks of intervention. WS root phytochemicals were identified by LC-ESI-MS. The inhibitory activity of these phytochemicals against angiotensin-converting enzyme 2 (ACE-2); the SARS-CoV-2 receptor; myeloperoxidase (MPO); and interleukin-6 (IL-6) was evaluated by *in silico* docking to investigate the mechanism of action of WS.

**Results:** The pulmonary functioning, quality of life, and exercise tolerance improved, and inflammation reduced notably the most in the WS group. Systemic oxidative stress subsided significantly only in the WS group. Although a minor placebo effect was observed in the SGRQ test, but it was not present in other tests. Withanolides found in the WS roots demonstrated substantial inhibitory activity against the proteins ACE-2, MPO, and IL-6, compared to that of a standard drug or known inhibitor. Moreover, FEV1% predicted had significant correlation with systemic antioxidative status (positive correlation) and malondialdehyde (MDA, negative correlation), suggesting that the antioxidative potential of WS has significant contribution to improving lung functioning.

**Conclusion:** Our study clinically demonstrated that WS root when given along with conventional drugs ameliorated COPD significantly more in comparison to the conventional drugs alone, in GOLD 2 and 3 categories of COPD patients. *In silico*, it has potent inhibitory activity against SARS-CoV-2 receptor, ACE-2, MPO, and IL-6.

## Introduction

COPD is a chronic pulmonary disease, characterized by partially reversible chronic airflow obstruction, inflammation, and small-airway remodeling. The WHO reported 251 million cases and 3.17 million deaths by COPD globally in 2015 (WHO fact sheet, 2021). Salvi et al. reported 30 million COPD cases in India (Salvi and Agrawal, 2012). It is estimated to be the third leading cause of death globally by 2030. It is one of the diseases included in WHO Global Action Plan for the Prevention and Control of Noncommunicable Diseases (NCDs). The risk factors include exposure to tobacco smoke, air pollution, and occupational pollution. COPD has no cure until now. Moreover, due to the increasing ambient air pollution (Shaddick et al., 2020) people are at higher risk of developing COPD (Andersen et al., 2011). Recent studies have reported that COPD patients are at a higher risk of severe outcomes of COVID-19 infection (Hansen et al., 2021). Numerous studies have demonstrated that increased oxidative stress and inflammation are the two major driving mechanisms in COPD progression (Oudijk et al., 2003; Kirkham and Barnes, 2013; McGuinness and Sapey, 2017).

Standard COPD medications, such as corticosteroids, only provide symptomatic relief, reduce further airway damage, and improve quality of life. They do not have disease-modifying efficacy. Moreover, corticosteroids have a number of adverse side effects, including osteoporosis, osteonecrosis, myopathy, increased risk of infections, edema, hypertension, and risk of cataract (Yasir et al., 2021). Thus, in the current scenario, a superior therapeutic strategy for COPD in clinical settings is the need of the hour.

*Withania somnifera (L.) Dunal [Solanaceae]* (Ashwagandha, the “Indian Ginseng”), also known as a “wonder herb”, is an adaptogen as shown by *in vivo* and clinical studies (Bhattacharya and Muruganandam, 2003; Chandrasekhar et al., 2012)*.* Adaptogens are unique plants that nonspecifically resist against diverse stressors and restore the systemic homeostasis (Brekhman and Dardymov, 2003) such as *Withania somnifera (L.) Dunal* [*Solanaceae*], *Sedum rosea (L.) Scop [Crassulaceae]*, and *Panax ginseng C.A. Mey* (*Araliaceae*)*.* Extensive studies have been carried out till now on WS, showing its diverse therapeutic properties, namely, immunomodulatory (both immunosuppressive and immunostimulatory), neuromodulatory, anticancer, cognition-promoting, antifungal, antibacterial, antidepressant, antiarthritic, and so on (Ghosal et al., 1989; Wagner et al., 1994; Bhattacharya et al., 1995). Even the WHO has recognized complementary medicine importance in disease treatment and has developed WHO Traditional Medicine Strategy 2014–2023 for working on traditional herbal medicine and its integration into national health systems.

SARS-CoV-2 is the virus that causes coronavirus-2019 disease (COVID-19), the current pandemic. As of March 2022, 46.6 crore confirmed cases and 60.7 lakh deaths by COVID-19 had been reported worldwide. Previous studies have reported a comparatively higher rate of hospitalization, ICU admission, and mortality by COVID-19 in COPD patients than non-COPD patients (Gerayeli et al., 2021). Other clinical studies also suggested that COPD does not increase COVID-19 susceptibility, but it increases the risk of severe outcomes. An upregulation of ACE-2, a receptor for SARS-CoV-2 entry, into the host epithelial cells of COPD patient’s airways could be one of the main reasons (Leung et al., 2020; Milne et al., 2020). Thus, inhibiting the ACE-2 receptor can be a worthwhile strategy for reducing the possibilities of severe outcomes by COVID-19 in COPD patients.

The objectives of the present study are as follows: 1) to study the add-on effect of WS on COPD patient’s pulmonary functioning, quality of life, and exercise tolerance; (2) to find its mechanism of action by evaluating its (a) antioxidant or anti-inflammatory potential; (b) correlating it with lung functioning parameters; and (c) *in silico* identification of potential inhibitors of MPO and IL-6 proteins; and (3) to identify the phytochemicals present in WS root extract and *in silico* analyzing their inhibitory activity against ACE-2, thus evaluating its antiviral potential.

## Materials required:

2,4,6-tris (2-pyridyl)-s-triazine (TPTZ) (catalog no. T1253), cetyltrimethylammonium bromide (CTAB) (catalog no. H6269), 3,3′,5,5′-tetramethylbenzidine (TMB) (catalog no. T2885), vanadium (III) chloride (catalog no. 208272), N-(1-naphthyl) ethylenediamine dihydrochloride (catalog no. 222488), sulfanilamide (catalog no. S9251), bovine serum albumin (BSA) (catalog no. A9418), thiobarbituric acid (catalog no. T5500), trichloroacetic acid (catalog no. 91230), ethylenediaminetetraacetic acid disodium salt dehydrate (EDTA-Na_2_) (catalog no. E4884), guanidine hydrochloride (GnHCl) (catalog no. G3272), and sodium nitrite (NaNO_2_) (catalog no. 237213) were purchased from Sigma. Thiobarbituric acid (catalog no. RM 1594), 2,4-dinitrophenylhydrazine (catalog no. RM 1059), 5,5′-dithiobis-2-nitrobenzoic acid (DTNB) (catalog no. GRM 1677), Wright-Giemsa (catalog no. S030 and TCL 083), and ferric chloride (FeCl_3_) (catalog no. GRM1379) were purchased from Himedia. IL-6 (catalog no. 950.030.192) and TNF-*α* (catalog no. 950.090.192) ELISA kits were purchased from Diaclone. All the other laboratory chemicals were of analytical grade.

## Methods

### Study Design

**FIGURE 1:**
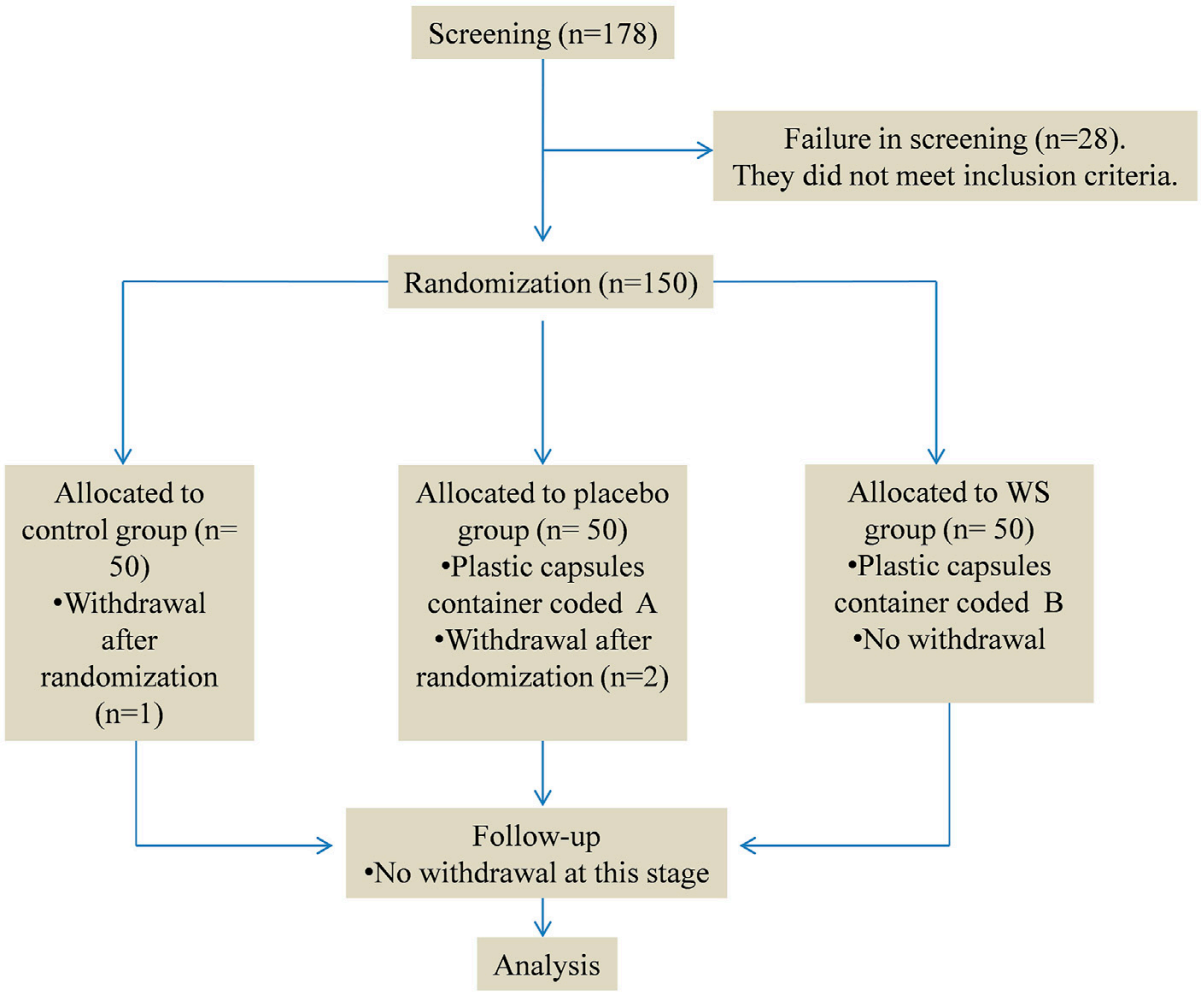
Study flowchart.

A randomized, placebo-controlled, and double-blind study was conducted at Jawaharlal Nehru Medical College, Aligarh Muslim University, Aligarh, Uttar Pradesh, India. This study was reviewed and approved by the ethical committee of Jawaharlal Nehru Medical College. There was a dearth of reference data from published research studies for the accurate sample size calculation. Thus, the sample size was determined by the institutional ethical committee and experiences of respiratory doctors. In June 2021, the first COPD patient was enrolled. The recruitment period lasted 5 months. A total of 105 COPD patients, meeting the inclusion criteria, were randomized into three groups (control group, placebo group, and WS group), with 50 patients in each group by using computer-generated random numbers. Simple randomization was carried out in the present study. A researcher who was not participating in the study saved the computer-generated randomization codes individually for each patient in a sealed envelope. Only when the patient met the inclusion criteria and was assigned a serial number, the doctors and researchers opened those envelops and came to know about the randomization codes. Three patients discontinued due to their busy schedules; one patient refused when knowing about methodology details. No patient dropped out during the follow-up period. The participant flowchart has been shown in Figure 1. The control group did not receive anything other than conventional medicines, that is, fluticasone propionate or prednisolone orally and long acting bronchodilator (Tiate or doxiflo) as needed. The placebo group received starch capsules along with conventional drugs. The WS group received WS root capsules along with conventional drugs for 12 weeks. COPD patients that were stable had no exacerbations or hospitalizations in the last 6 weeks were selected for the study. They were classified on the basis of severity according to the GOLD guidelines. Their blood samples were taken for further analysis at baseline and after 12 weeks of therapy.

A low and safe dose of WS root capsules was finalized based on the previous clinical studies showing no adverse side effects. The ayurvedic recommended dose of WS by the Department of Ayush, Ministry of Health and Family Welfare, Government of India is 3.0–6.0 g/day (Government of India, Ministry of AYUSH, 2016). Moreover, many studies have shown that 600 mg or more daily WS dose is safe and showed no adverse side effects (Sharma et al., 2018; Verma et al., 2021).

### Medicine Preparation, Double-Blinding, and Intervention

*WS* roots were purchased from an authorized dealer. The roots were then authenticated by experts from the Department of Botany, Aligarh Muslim University. They were shade-dried and ground into a fine powder using a laboratory grinder under aseptic conditions. *WS* root powder capsules (for the WS group) and starch capsules (for the placebo group) were produced by a Good Manufacturing Practice (GMP)–certified facility. Hard gelatin capsules were filled with 250 mg of either *WS* root powder or inert substance, starch. Both types of capsules were stored in a specific way to ensure the spread of smell of herb to placebo. These capsules were stored in coded plastic containers with silica gel at room temperature. The *WS* root powder and starch capsules and containers were all the same shape, size, and color; thus, they were indistinguishable. As a result, they could not be identified as a drug or a placebo. A researcher who was not involved in the study assigned distinct codes, A and B, to the drug and placebo capsule containers. These codes were only revealed after the study was completed, and the data were being analyzed. In this manner, double-blinding was ensured. In the present study, the WS group received 250 mg of WS root capsules orally twice per day, for an intervention period of 12 weeks. For quality consistency of the herb, all of the capsules needed for the entire study were prepared at the same time, aliquoted, and packed. All the patients were followed up regularly by researchers and nurses with the help of calls and SMS given on weekly basis to assure compliance with the treatment course. Medication use was also recorded.

### Inclusion Criteria

Inclusion criteria included

1) FEV1% predicted >30% and <80% for COPD.
2) Patient’s age is between 40 and 70 years.
3) Patients and their families understood the study protocol and gave their consent for participation.
4) Stable COPD of GOLD stage 2, 3.
5) Current or former smokers.
6) Receiving conventional drug therapy from not more than a month.

### Exclusion Criteria

Key exclusion criteria were the presence of acute respiratory tract infections within past 1 month, coronary heart disease, diabetes, lung cancer, severe hypertension, renal or hepatic failure, peptic ulcer, severe exacerbation, or hospitalization within past 4 weeks since this may interfere with the results. Also, pregnant and lactating women were excluded from the study.

### Clinical Evaluation

#### Pulmonary Function Test (PFT)

Pre and post treatment, FEV_1_ (forced expiratory volume in 1 s) % predicted, FVC (forced vital capacity) % predicted, and FEV_1_/FVC were measured by spirometry to evaluate lung functioning. These parameters were automatically calculated on the basis of height, sex, age, and ethnicity. The reading was taken in triplicates, and the best values were taken for each parameter.

#### St George’s Respiratory Disease Questionnaire for COPD (SGRQ-C)

It has 14 questions with multiple subquestions to evaluate the quality of life of COPD patients. It has three components, namely, 1) symptoms, 2) activity, and 3) impact. The score can vary from 0 to 71 score. The higher the score, the poorer is the quality of life. To avoid any chances of bias, the questionnaire was filled before PFT. Jones, the author of the questionnaire, considered four unit decrease as the minimal clinically significant reduction (Meguro et al., 2007).

#### 6-Minute Walk Test (6MWT)

For evaluating exercise tolerance and lung functioning, 6MWT was performed according to the standards of the American Thoracic Society (ATS) (Holland et al., 2014). Briefly, the patients had to walk on a flat ground for 6 min as far as they can.

### Antioxidant Studies

#### Total Antioxidant Capacity (FRAP Assay)

Antioxidant capacity of the plasma samples was determined using the ferric reducing antioxidant power (FRAP) assay. In the FRAP assay, antioxidants present in the samples reduces Fe^3+^ to Fe^2+^, which then reacts with 2,4,6-tris (2-pyridyl)-s-triazine (TPTZ), giving a blue colored complex, read at 593 nm. Ferrous sulfate was used as the standard. Thus, the antioxidant status of plasma samples was analyzed using a ferrous sulfate standard curve (Figure 5E) (Benzie and Strain, 1996).

#### MDA Assay

MDA, a stable end product of lipid peroxidation in plasma, was evaluated as a pink-colored product, thiobarbituric acid reactive substance (TBARS) using thiobarbituric acid. Its absorbance was read at 535 nm. The molar extinction coefficient of 1.56 × 10^5^ M^−1^cm^−1^ was used to determine the TBARS concentration. (Buege and Aust, 1978).

#### Protein Carbonyl Measure

Oxidative damage to proteins was assessed by quantifying the protein carbonyl group using 2,4-dinitrophenylhydrazine. This reaction yields dinitrophenylhydrazone adduct whose absorbance was read at 360 nm and molar extinction coefficient is 22 × 10^3^ M^−1^cm^−1^. The total protein content in plasma was evaluated using the Lowry method to determine the protein carbonyl content per milligram of protein. A bovine serum albumin (BSA) standard curve was developed and used to analyze the total plasma protein concentration (Figure 5F). (Levine et al., 1990).

#### Total SH Group

The total sulfhydryl groups present in plasma were measured spectrophotometrically by using 5,5′-dithiobis-2-nitrobenzoic acid (DTNB). It gets reduced by sulfhydryl groups to a colored product, thionitrobenzoate, having the absorption at 412 nm and molar extinction coefficient of 13,100 M^−1^cm^−1^ (Sedlak and Lindsay, 1968).

### Anti-Inflammatory Studies

#### Neutrophil Counting by Using Wright-Giemsa Staining

The patient’s blood was collected in EDTA tubes. Neutrophil counting was carried out by using the Wright–Giemsa staining. The slides were observed under a microscope at 60 × magnification. More than 100 cells per slide were counted. This was carried out to observe any alteration in the systemic neutrophil cell count.

#### MPO Activity Assay

After centrifugation, the buffy coat forming leukocytes were isolated from blood, followed by counting by a hemocytometer and disruption using cetyltrimethylammonium bromide (CTAB) detergent (Pulli et al., 2013). Its supernatant was used to quantify peroxidase enzyme according to Marquez and Dunford, (1997). 3,3′,5,5′-tetramethylbenzidine (TMB) donates hydrogen to hydrogen peroxide and forms water and a blue colored product 3,,3′,5,5′-tetramethylbenzidine diimine, a reaction catalyzed by peroxidase enzyme. Its absorbance was read at 652 nm for 5 min. For quantifying TMB oxidation product, an extinction coefficient of 3.9 × 10^4^ M^−1^ cm^−1^ was used (Josephy et al., 1982). Enzyme activity was expressed in µM/mg protein in a single leukocyte.

#### Nitric Oxide Assay

Nitric oxide (NO) is very unstable and gets oxidized to stable metabolites, nitrite and nitrate. So, first by using vanadium (III) chloride, nitrate was reduced to nitrite, and then nitrite concentration was measured by using the Greiss reagent [0.1% N-(1-naphthyl) ethylenediamine dihydrochloride and 1% sulphanilamide] at 540 nm. Here, sodium nitrite was used as a standard. Its standard curve was used to evaluate the NO level in plasma samples, and it is shown in Figure 6G (Miranda et al., 2001).

#### ELISA

IL-6 and TNF-*α* were determined in the patient’s serum samples by using ELISA kits, according to the manufacturer’s instructions.

#### LC-ESI-MS

For analyzing WS root phytochemicals, its methanolic extract was prepared by the soxhlation method. This was followed by its analysis by XEVO-using a TQD mass spectrometer. Capillary voltage was kept 3.5 kV, cone voltage: 30–34.92 V, source temperature: 120°C, and dissolvation temperature: 350°C.

#### Docking

The crystal structures of proteins, namely, ACE-2, MPO, and IL-6 (PDB ID: 1R4L, 1DNU, and 1ALU, respectively) were collected from RCSB Protein Data Bank. The 3-D structures of all the ligands were retrieved from https://pubchem.ncbi.nlm.nih.gov. Hydroxychloroquine (standard drug of SARS-CoV-2 and known ACE-2 inhibitor), L-tyrosine (known MPO substrate), and methotrexate (known IL-6 inhibitor) were taken as standards. Molecular docking was performed using Autodock-vina. Protein structures were prepared for docking using Chimera 1.10.2. The size of the grid was set at 30–30–30⁰A, and the centre of the grid at x: 43.841, y: 3.753, z: 20.164 (ACE-2); x: 22.172, y: −17.332, z: 1.891 (MPO); x: −2.827, y: −22.930, z: −1.448 (IL-6). The images were prepared using PyMOL Molecular Graphics System.

#### Statistical Analysis

The data were expressed as the mean ± SD. The results were statistically analyzed by two-way ANOVA with post-hoc Tukey test, and the results are included in their respective graphs and shown in Table 1. The data with *p* < 0.05 were considered statistically significant. The present study did not include any interim analyses.

**TABLE 1:** Two-way ANOVA results of all the clinical parameters evaluated.

Two-way ANOVA results	Groups	Time	Interaction
Parameters FEV1% predicted	<0.001	<0.001	0.020
FVC % predicted	ns	<0.001	ns
FEV1/FVC % predicted	<0.001	<0.001	0.010
SGRQ score	0.008	<0.001	ns
6MWT	<0.001	<0.001	<0.001
FRAP assay	<0.001	<0.001	<0.001
MDA assay	<0.001	<0.001	<0.001
Protein carbonyl content	<0.001	<0.001	<0.001
Total SH group	<0.001	<0.001	<0.001
Neutrophil count	0.005	<0.001	<0.001
MPO activity	<0.001	<0.001	<0.001
Nitric oxide assay	0.014	<0.001	0.006
TNF-alpha	ns	ns	ns
IL-6	ns	<0.001	ns
IL-6 (GOLD2)	0.032	<0.001	0.004
IL-6 (GOLD3)	ns	<0.001	ns

## Results

### Demographic Data

There was no significant difference in treatment regimens and baseline characteristics among the three groups (Table 2).

**TABLE 2:** Baseline characteristics of the subjects.

	Control group (*n* = 49)	Placebo group (*n* = 48)	WS group (*n* = 50)
Age (years)	56.18 ± 7.33	58.97 ± 8.56	58.75 ± 7.34
Gender (male/female)	30/19	26/22	24/26
BMI (kg/m^2^)	21.86 ± 1.92	22.1 ± 1.81	22.88 ± 2.49
Pulmonary function			
FEV1 (% predicted)	46.18 ± 10.72	47.39 ± 8.44	48.34 ± 9.11
FVC (% predicted)	49.22 ± 10.48	51.88 ± 7.63	52.6 ± 8.65
FEV1/FVC (% predicted)	93.47 ± 2.91	91.25 ± 7.30	91.6 ± 4.4
Former/current smokers	32/17	37/11	29/21
GOLD stage			
II	19	22	23
III	30	26	27

### Primary Outcomes

#### Pulmonary Function Test

**FIGURE 2:**
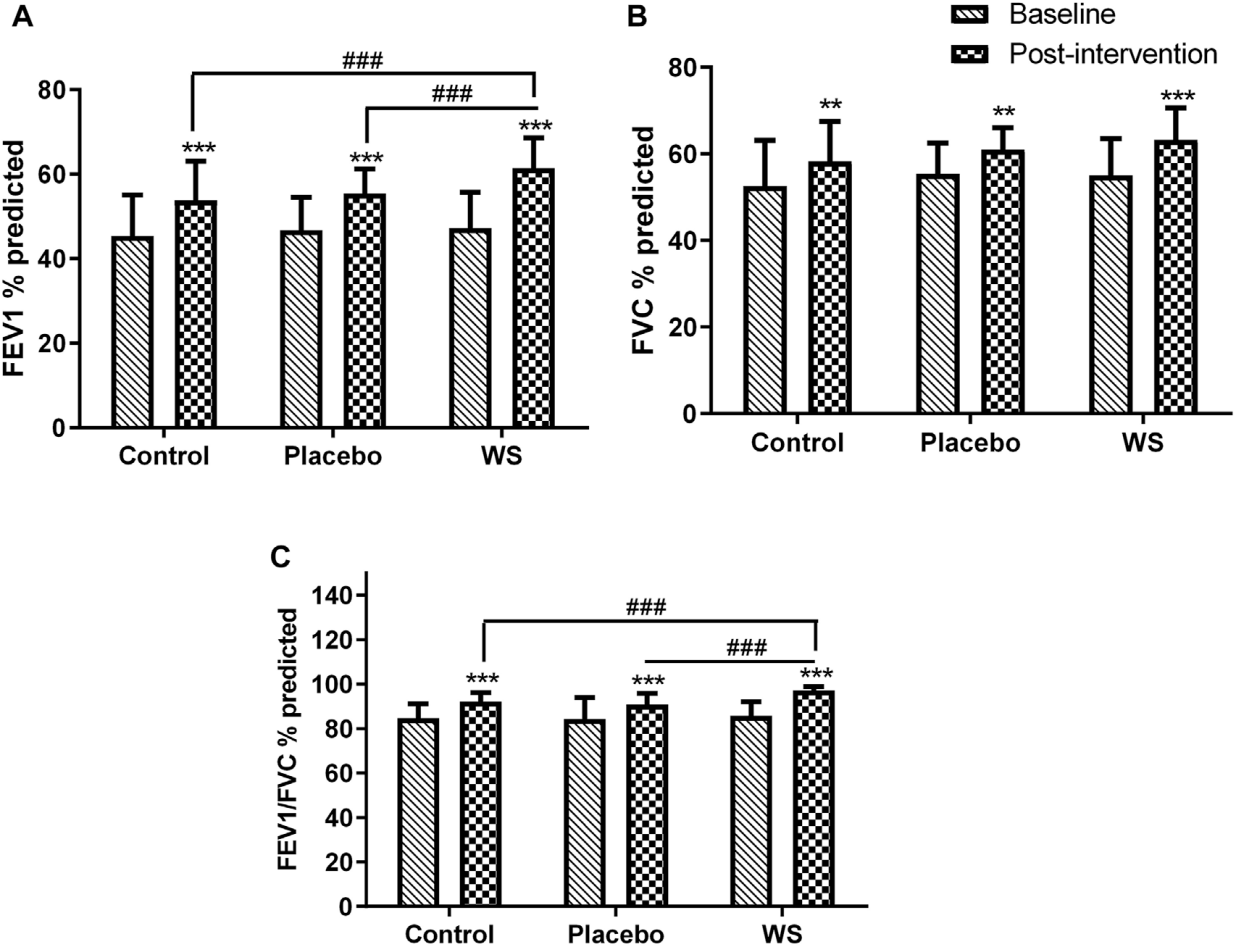
Lung function before and after intervention: Mean **(A)** FEV_1_% predicted, **(B)** FVC % predicted, and **(C)** FEV_1_/FVC % predicted values at the baseline and at the end of the study. *p ≤ 0.05, **p ≤ 0.01, and ***p ≤ 0.001 compared with their respective baselines. ^###^ *p* ≤ 0.001.

There was no significant difference in the baseline values of the pulmonary function test of the three groups. After 12-week intervention period, FEV_1_% predicted (Figure 2A), FVC % predicted (Figure 2B), and FEV_1_/FVC ratio (Figure 2C) got increased significantly in all the three groups. In the WS group, it increased by 14.16, 8.2, and 11.38%; in the control group, it is 8.37, 5.66, and 7.47%; and in the placebo group, it has increased by 8.695, 5.54, and 6.45%, respectively, from their respective baselines. There was a significant difference in the post-intervention values of FEV_1_% predicted and FEV_1_/FVC of control (7.69 and 4.97%, respectively) and placebo groups (6.05 and 6.36%, respectively) from the WS group. But, there was no significant difference in the post-intervention PFT values of control from the placebo group. Thus, WS intervention had significantly improved lung functioning in comparison to only the conventional drug or conventional with placebo capsule intervention. Also, the PFT values did not show any placebo effect.

#### SGRQ-C Score

**FIGURE 3:**
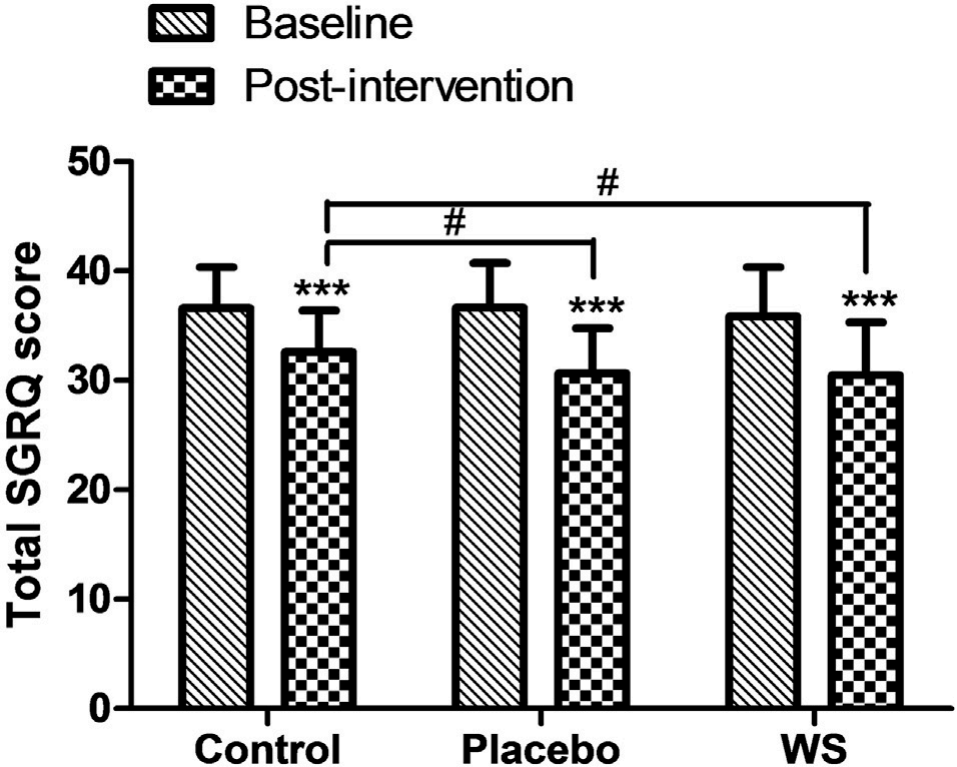
Effect of interventions on the quality of life of patients: Mean SGRQ total score at the baseline and at the end of the study. ***p ≤ 0.001 compared with baseline. ^#^ *p* ≤ 0.05.

Postintervention, the SGRQ-C score decreased in all the three groups significantly, but it was more significant in the WS (35.84 ± 4.54 to 30.48 ± 4.58 i.e., −5.36) and placebo group (36.62 ± 3.76 to 30.66 ± 4.03 i.e., −5.96) than the control group (36.61 ± 3.71 to 32.59 ± 3.88 i.e., −4.02) (Figure 3). Moreover, responders %, that is, percent of COPD patients with at least four unit minimum decrease in SGRQ-C score after the completion of study was much higher in the WS (80%) and placebo group (77.08%) than in the control group (53.06%) (Supplementary Figure S1). Thus, it was similar in the WS and placebo groups, indicating placebo effect.

#### 6MWT

**FIGURE 4:**
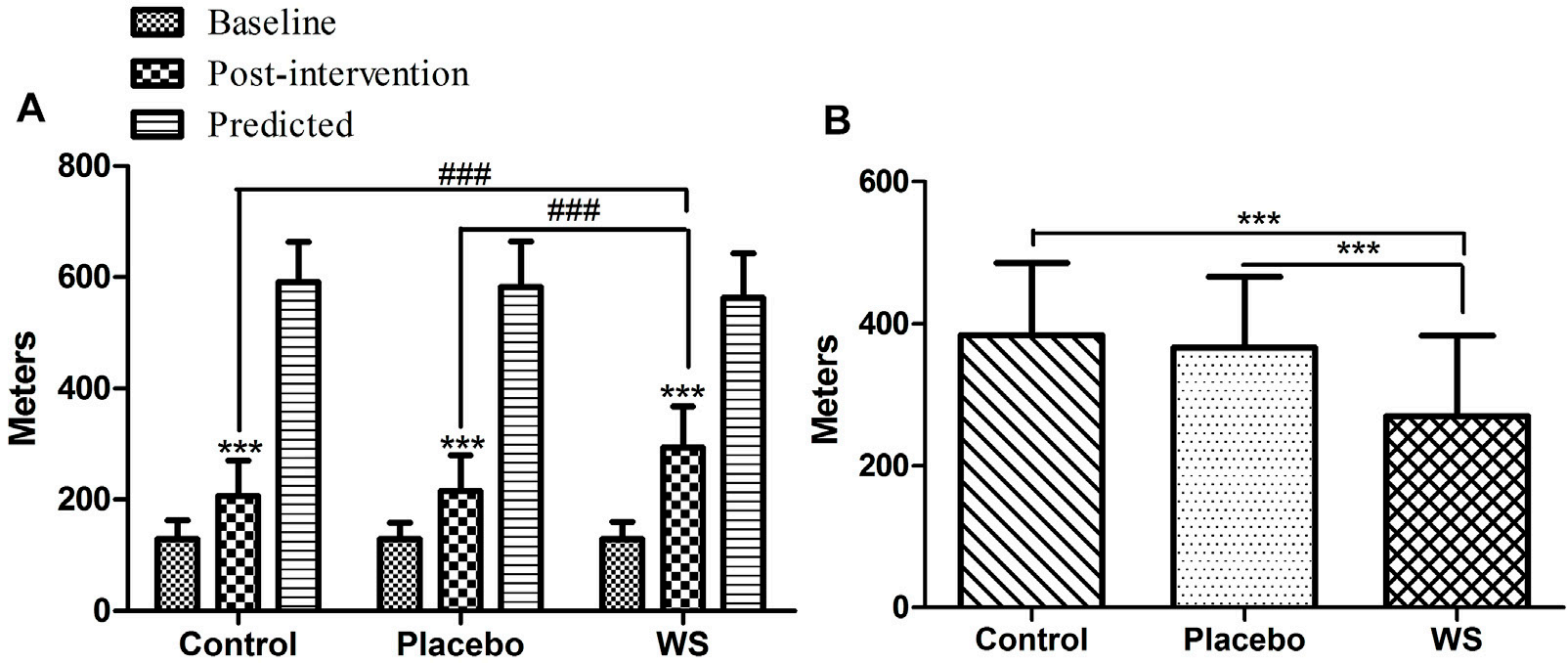
Effect of interventions on exercise tolerance and lung functioning, measured by 6MWT: **(A)** Mean distance walked during 6 MWT (in meters) at the baseline and at the end of the study. **(B)** Difference of post-intervention 6MWT value from predicted values ***p ≤ 0.001 compared with baseline. ^###^ *p* ≤ 0.001.

After 12-week intervention period, 6 min walk distance improved significantly in all the three groups (WS group: 129.04 ± 31.06 m to 293.48 ± 73.48 m, control group: 129.63 ± 33.42 m to 206.86 ± 63.77 m, and placebo group: 129.63 ± 29.04 m to 215.46 ± 64.42 m), but it was the highest in the WS group (Figure 4A). Moreover, the mean difference of evaluated 6MWT post-intervention scores from predicted 6MWT scores decreased mostly in the WS group (control group: 384.45 m, placebo group: 366.66 m, and WS group: 269.62 m) (Figure 4B).

### Secondary Outcomes

#### Antioxidant Status

**FIGURE 5:**
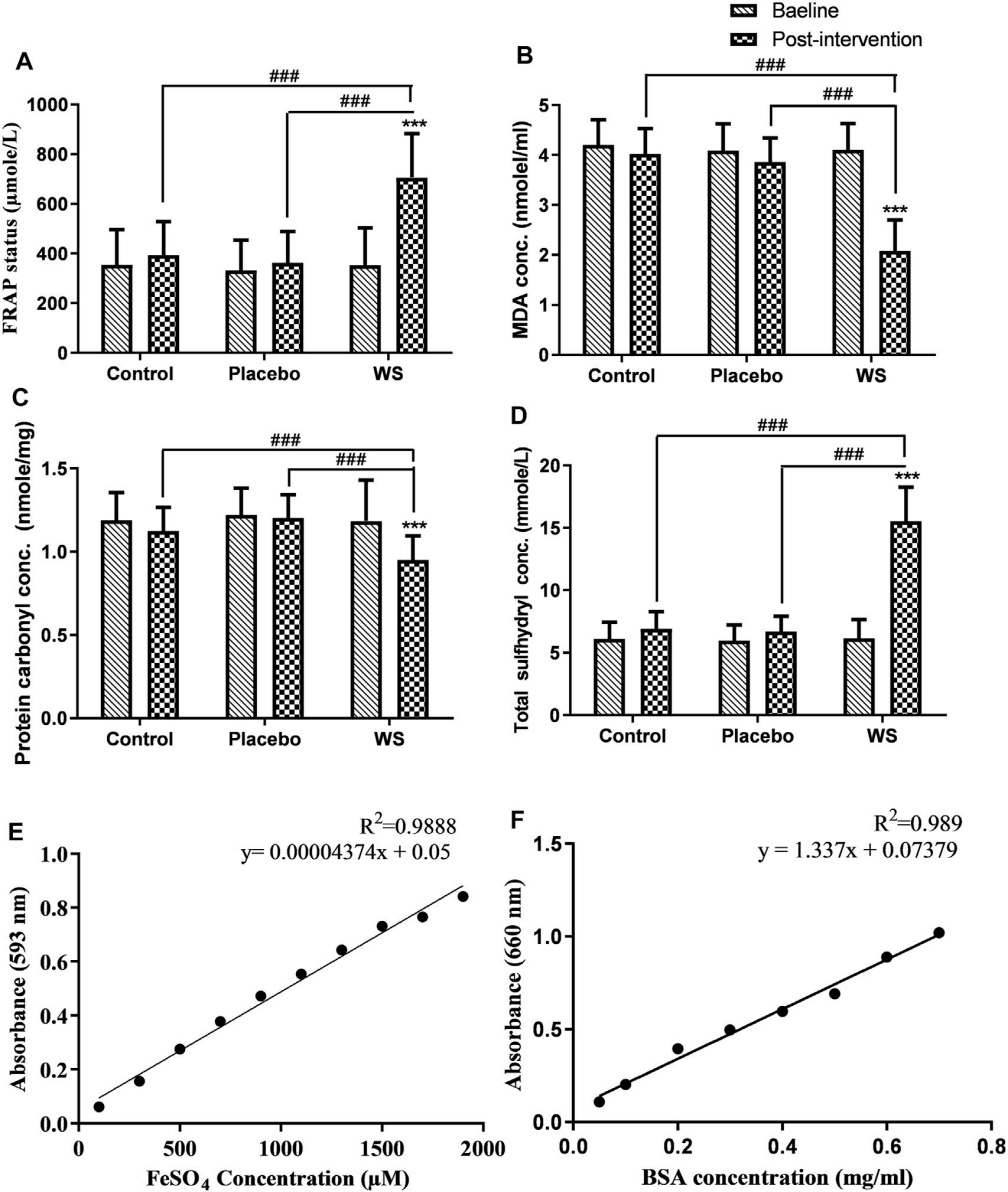
Effect of interventions on systemic OS of COPD patients: Mean **(A)** total antioxidant status (FRAP status), **(B)** MDA concentration, **(C)** protein carbonyl concentration, **(D)** total sulfhydryl concentration in plasma at the baseline and at the end of the study, **(E)** Standard curve of ferrous sulfate concentration, **(F)** Standard curve of BSA protein concentration. ***p ≤ 0.001 compared with baseline. ^###^ *p* ≤ 0.001.

Antioxidant status, as measured by the FRAP assay was poor in COPD patients at the baseline. After 12 weeks of treatment, it improved in the WS group (352.9 ± 149.67 µMole/L to 705.22 ± 178 µMole/L) with high significance in comparison to that of the control (354.86 ± 141.03 µMole/L to 392.56 ± 135.67 µMole/L) or placebo (332.32 ± 122.14 µMole/L to 362.53 ± 126.43 µMole/L) groups (Figure 5A). Moreover, the antioxidant status and FEV_1_% predicted had significant positive correlation (Table 3).

**TABLE 3:** Pearson’s correlation of FEV1% predicted values with FRAP status and MDA concentration.

Parameters	FRAP status	MDA conc.
FEV1% predicted		
r	0.7516	−0.73
*p*	<0.0001	<0.0001

#### Lipid Peroxidation Status

A highly significant decrease was observed in the post-intervention plasma MDA concentration in the WS group (4.11 ± 0.52 nmol/ml to 2.08 ± 0.62 nmol/ml) in comparison to baseline and post-intervention values of the control (4.2 ± 0.5 nmol/ml to 4.02 ± 0.51 nmol/ml) and placebo (4.09 ± 0.54 nmol/ml to 3.86 ± 0.48 nmol/ml) groups (Figure 5B). Also, it had a significant negative correlation with FEV_1_% predicted values (Table 3).

#### Protein Carbonyl Content

A similar pattern was shown by the plasma protein carbonyl content, that is, a highly significant decrease was observed post intervention in the WS group (1.18 ± 0.24 nmol/mg protein to 0.95 ± 0.14 nmol/mg protein) in comparison to its baseline and post-intervention values of the control (1.19 ± 0.17 nmol/mg protein to 1.12 ± 0.14 nmol/mg protein) and placebo (1.22 ± 0.16 nmol/mg protein to 1.2 ± 0.14 nmol/mg protein) groups (Figure 5C). But, it had no significant correlation with FEV_1_% predicted values (data not shown).

#### Total Sulfhydryl Concentration

In the present study, total sulfhydryl concentration in the plasma of COPD patients was significantly low at the baseline. Although it increased in all the three groups after 12 weeks of intervention, its concentration was highest in the WS group (WS group: 6.16 ± 1.48 mmol/L to 15.55 ± 2.71 mmol/L; control group: 6.1 ± 1.36 mmol/L to 6.9 ± 1.4 mmol/L; and placebo group: 6 ± 1.25 mmol/L to 6.70 ± 1.21 mmol/L) (Figure 5D).

#### Neutrophil Count

**FIGURE 6:**
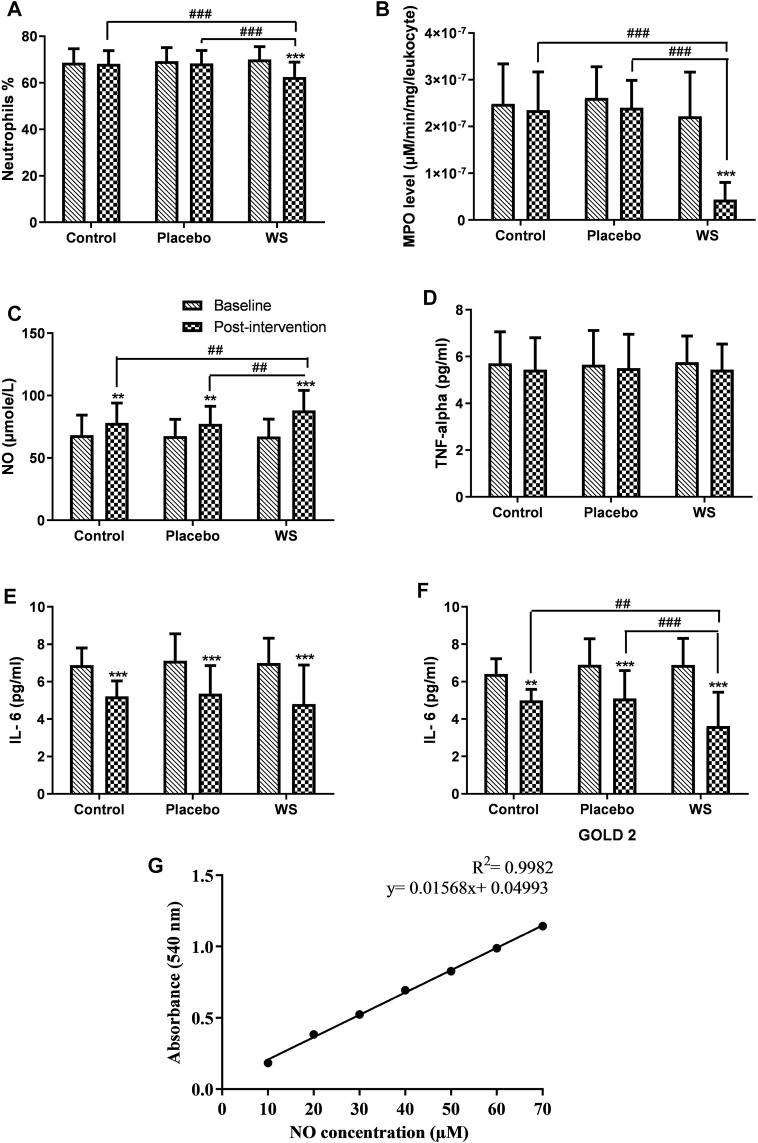
Effect of interventions on systemic inflammation of patients: Mean **(A)** neutrophils %, **(B)** MPO level (in blood leukocytes), **(C)** NO concentration, **(D)** TNF-alpha, **(E)** IL-6 (GOLD 2 and 3 combined), **(F)** IL-6 (GOLD 2 category patients) in blood of patients at the baseline and at the end of the study, **(G)** Standard curve of NO concentration. **p ≤ 0.01, ***p ≤ 0.001, compared with baseline. ^##^ *p* ≤ 0.01, ^###^ *p* ≤ 0.001.

After 12 weeks of intervention period, the neutrophil count decreased significantly only in the WS group (70.02 ± 5.47% to 62.5 ± 6.4%) (Figure 6A).

#### Myeloperoxidase Activity

Myeloperoxidase activity per leukocyte was found to be higher in COPD patients. Post intervention, an extremely significant decrease in its activity was observed in the WS group in comparison to the other two groups (control group: 2.48 × 10^−07^ ± 8.57×10^−08^ μM/min/mg/leukocyte to 2.35 × 10^−07^ ± 8.14×10^−08^ μM/min/mg/leukocyte, placebo group: 2.61 × 10^−07^ ± 6.68×10^−08^ μM/min/mg/leukocyte to 2.4 × 10^−07^ ± 5.86×10^−08^ μM/min/mg/leukocyte, and WS group: 2.22 × 10^−07^ ± 9.42×10^−07^ μM/min/mg/leukocyte to 4.35 × 10^−08^ ± 3.7×10^−08^ μM/min/mg/leukocyte) (Figure 6B).

#### NO Assay

NO level in the blood was found to be in the normal range, but it was at the higher side of it. After 12 weeks of intervention period, its level increased in all the three groups significantly. Its increase was significantly higher in the WS group (67.24 ± 13.82 μM to 88.1 ± 16.07 μM) than the control (68.20 ± 16.16 μM to 78.01 ± 16.06 μM) and placebo groups (67.46 ± 13.41 μM to 77.34 ± 14.08 μM) (Figure 6C).

#### ELISA

In COPD patients, serum IL-6 and TNF-*α* were both found to be increased significantly. In all the three groups, the TNF-*α* level decreased but nonsignificantly (Figure 6D), whereas IL-6 decreased significantly in all the three groups, but there was no significant differences in their post-intervention values (WS group: 6.99 ± 1.33 pg/ml to 4.8 ± 2.08 pg/ml, control group: 6.88 ± 0.93 pg/ml to 5.21 ± 0.82 pg/ml, and placebo group: 7.11 ± 1.44 to 5.35 ± 1.5 pg/ml) (Figures 6E,F). Interestingly, in GOLD 2–category patients, there was a more significant reduction in the WS group (6.88 ± 1.42 pg/ml to 3.62 ± 1.81 pg/ml) than the other two groups. But, that was not the case with GOLD 3 category patients.

#### LC-ESI/MS

**FIGURE 7:**
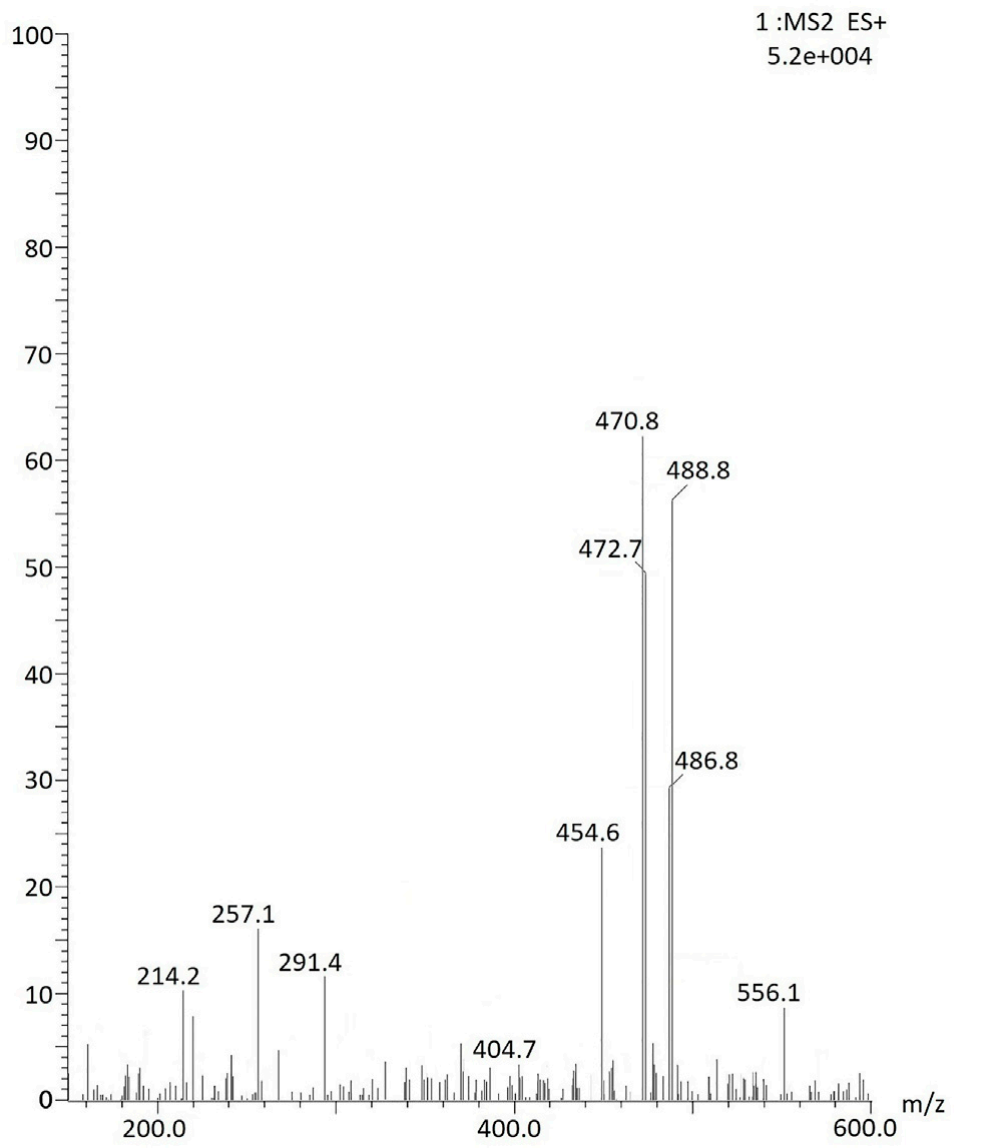
LC-ESI/MS spectrum of WS root extract at t_R_ 25.04 min.

The WS root extract mass spectrum at time t_R_ 25.04 min data analysis provides evidence for the presence of withanolides 1-8 in Table 4. The withanolides 1–4 and 7 had molecular ion peak at m/z 470.8 [M + H]^+^ (calc. C_28_H_39_O_6_, 470.8) and fragmented molecular ion peak at m/z 488.8 [M + NH4]^+^ (calc. C_28_H_42_O_6_N, 488.8). Withanolides 6, 5, and 8 had a molecular ion peak at m/z 472.7 [M + H]^+^ (calc. C_28_H_40_O_6,_ 472.7), 486.8 [M + H]^+^ (calc. C_28_H_38_O_7,_ 486.8), and at m/z 454.6 [M + H]^+^ (calc. C_28_H_38_O_5,_ 454.6), respectively (Figure 7; Table 4).

**TABLE 4:** Withanolides proposed to be present in WS root extract, on the basis of their m/z.

S. No.	Phytochemicals	m/z
1	Withanone	470.8 (and 488.8)
2	Withanolide A	470.8 (and 488.8)
3	Withaferin A	470.8 (and 488.8)
4	Withanolide D	470.8 (and 488.8)
5	Withasomniferol A	486.6
6	Withasomniferol B	472.7
7	Withasomniferol C	470.8 (and 488.8)
8	27-Deoxywithaferin A	454.6

#### Docking

**FIGURE 8:**
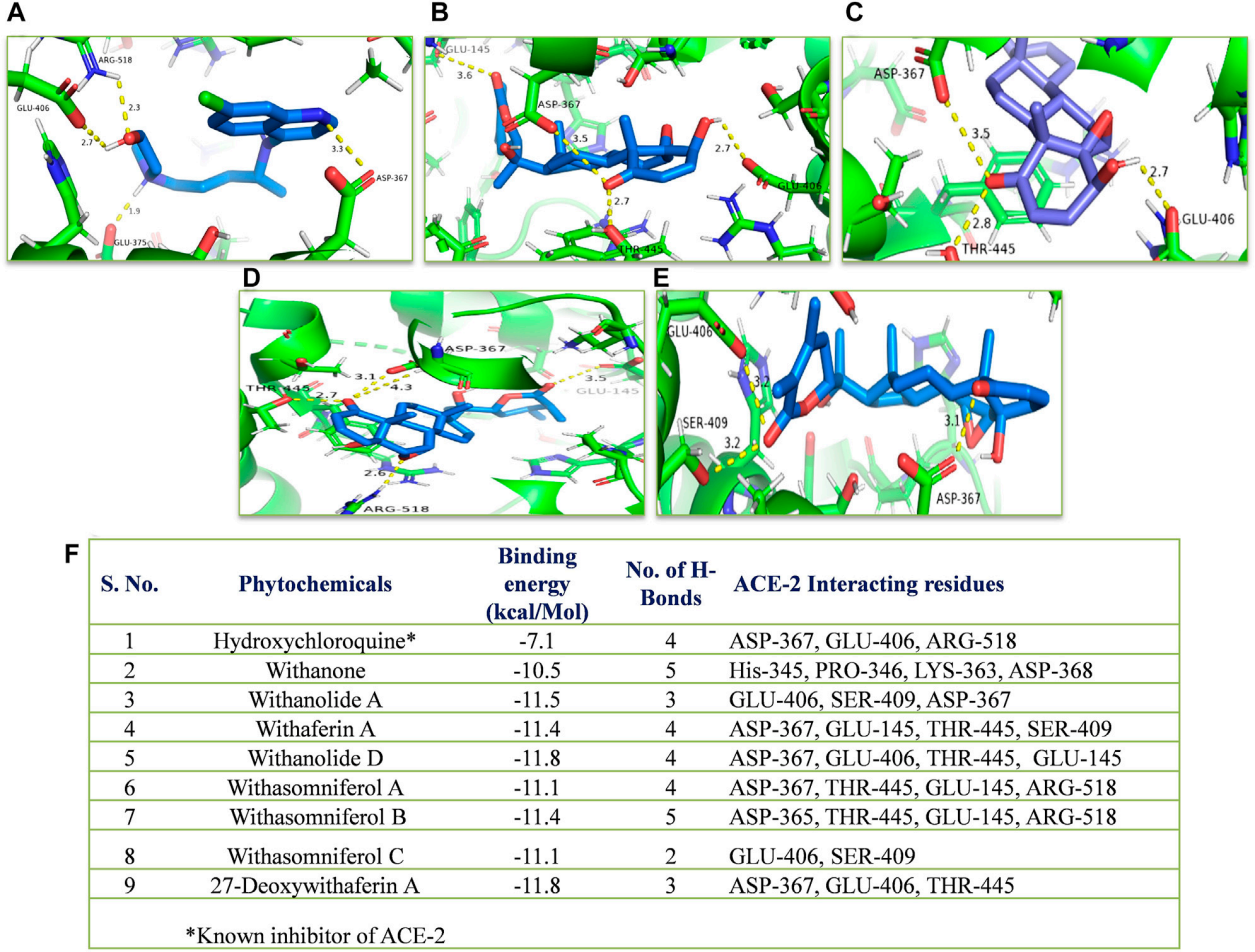
Molecular docking of the proposed withanolides with ACE-2. Docked conformations of withanolides and standard drug [**(A)** hydroxychloroquine, **(B)** withanolide D, **(C)** 27 deoxy withaferin, **(D)** withasomniferol B, **(E)** withanolide A] within the ACE-2 drug-binding cavity. **(F)** molecular docking results table. The protein interacting residues are labeled (in black) with the H-bond interactions (represented by yellow dashed lines).

**FIGURE 9:**
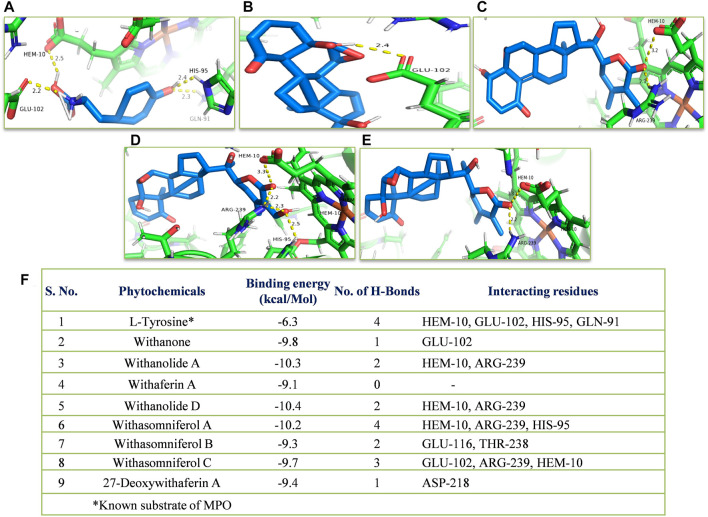
Molecular docking of the proposed withanolides with MPO. Docked conformations of withanolides and substrate **(A)** tyrosine, **(B)** withanone, **(C)** withanolide D; **(D)** withasomniferol A, **(E)** withanolide **(A)** within the MPO substrate-binding cavity; **(F)** molecular docking results table. The protein interacting residues are labeled (in black) with the H-bond interactions (represented by yellow dashed lines).

**FIGURE 10:**
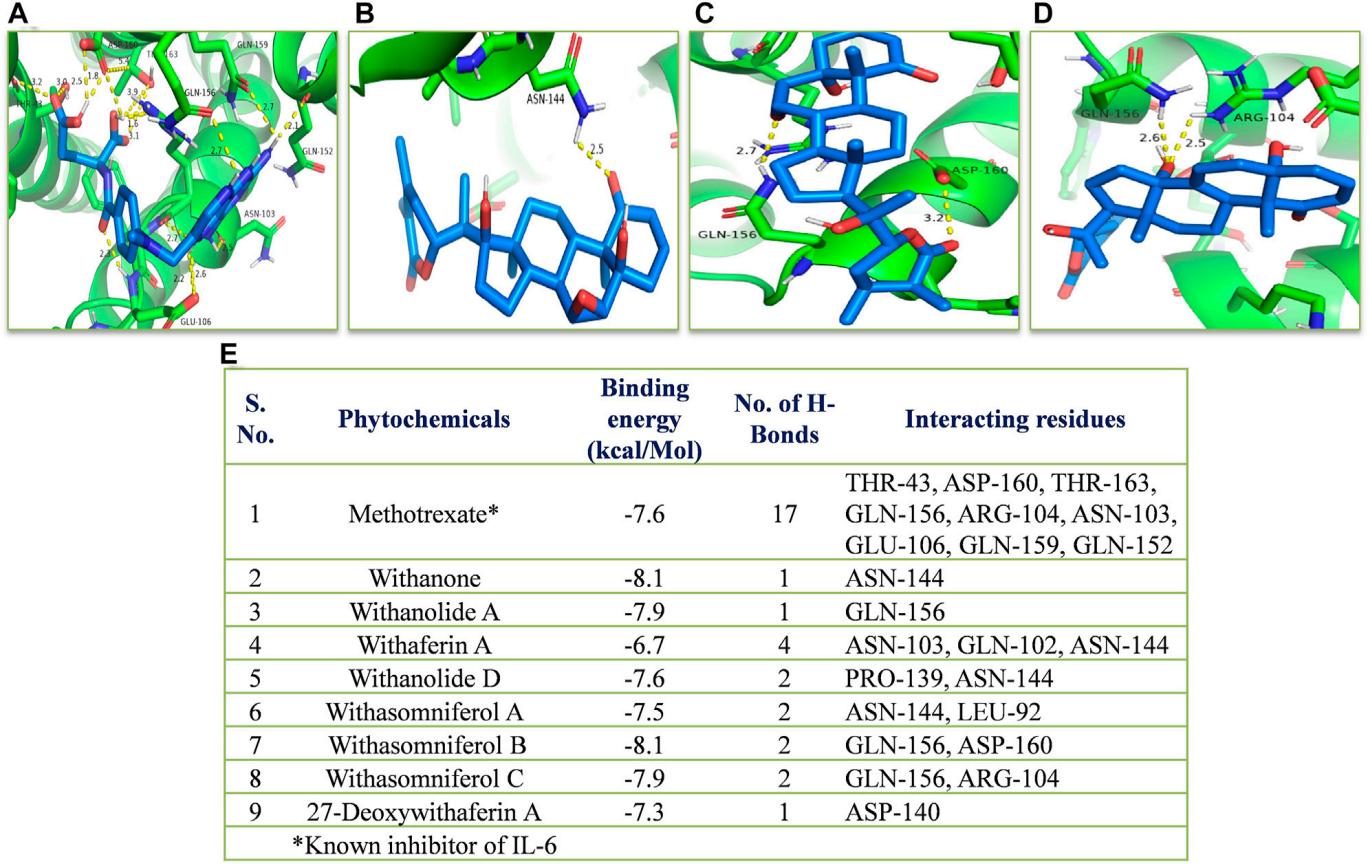
Molecular docking of the proposed withanolides with IL-6. Docked conformations of withanolides and known inhibitor **(A)** methotrexate, **(B)** withanone, **(C)** withasomniferol B, **(D)** withasomniferol **(C)** within the IL-6 inhibitor-binding cavity; **(E)** molecular docking results table. The protein interacting residues are labeled (in black) with the H-bond interactions (represented by yellow dashed lines).

*In silico*, all the eight withanolides showed strong ACE-2 inhibitory activity with the binding energy ranging from −11.8 to −10.5 kcal/Mol. Withanolide D and 27-deoxywithaferin A had the strongest binding energy of −11.8 kcal/Mol for ACE-2, much stronger than the standard drug, hydroxychloroquine binding energy, −7.1 kcal/Mol. Similarly, withanolides showed good binding energy for MPO and IL-6. Among these withanolide D had the strongest binding energy, that is, −10.4 kcal/Mol for MPO; and withasomniferol B and withanone had the strongest binding energy of value −8.1 kcal/Mol for IL-6 protein, while a known inhibitor, methotrexate has −7.6 kcal/Mol binding energy. Figures 8–10 shows the number of H-bonds formed between proteins and ligands and the interacting residues of proteins for various ligands.

## Discussion

Previously, many medicinal plants and their compounds, namely, *Nigela sativa* (Salem et al., 2017)*, Curcuma longa* (Manarin et al., 2019), pomegranate juice (Cerdá et al., 2006), ginseng (Gross et al., 2014), curcumin (Kim et al., 2000), and many others have been studied clinically for inflammatory lung disease, but herbs having adaptogenic nature and steroidal compounds have not been evaluated yet clinically for obstructive lung disease. With the passage of time, an increasing number of studies on adaptogens have revealed that they have multiple targets, implying that it is still worthwhile to exploit adaptogens for other diseases as well. WS is adaptogenic and has numerous bioactive steroidal compounds. Previous studies of WS showed its anti-inflammatory and antioxidative properties. *In vivo*, it increased the WBC count, RBC count, hemoglobin, phagocytic activity of macrophages, and antibody production from spleen lymphocytes (Ziauddin et al., 1996; Yamada et al., 2011). *In vivo* and clinical studies revealed that it reduces stress and its related cytokines, namely, IFN-*γ*, IL-6, and IL-12 (Vetvicka and Vetvickova, 2011). It has been studied on mice asthma model where it decreased the white blood cell count both in the bronchoalveolar lavage and blood, suggesting its potential anti-inflammatory properties (Oberholzer et al., 2008). This is the first study where WS has been studied for COPD patient treatment. In the present study, we evaluated the add-on effect of WS with the conventional medicines on lung functioning, quality of life, and exercise tolerance of COPD patients. Since, oxidation and inflammation are known important pathophysiological factors of COPD, we also studied the potential antioxidation and anti-inflammatory effects of WS on COPD patients.

The LC-ESI-MS mass spectrum provides evidence for the presence of bioactive steroids (withanolides), namely, withanone, withanolide A, withaferin A, withanolide D, withasomniferol A, withasomniferol B, withasomniferol C, and 27-deoxywithaferin A in WS roots used by us in our study. After a 12-week intervention period, the WS group’s FEV1% predicted and FEV1/FVC % predicted values were found to be significantly better than those of the control or placebo groups, indicating that WS was an add-on therapy enhanced lung function. This is well-known that the quality of life is impaired in COPD patients. SGRQ and 6MWT were used to analyze it. SGRQ decreased and 6MWT increased the best in the WS group, indicating a significant improvement in the quality of life of patients by WS. Moreover, in the placebo group, SGRQ score decreased more than that of the control group, indicating a placebo effect. Since it was based on a questionnaire, chances of getting placebo effect here is more, but since any other lung test or systemic parameter did not show similar results; thus, we can conclude that the overall results in the WS group are not caused due to the placebo effect.

Chronic inflammation and OS are the two crucial controlling mechanisms of COPD pathogenesis and disease progression. Exogenous oxidants, namely, cigarette smoke and air pollution are the primary sources of elevated OS systemically and in the lungs of COPD patients. Numerous studies have shown the manifestation of systemic OS and inflammation in COPD patients (Rahman et al., 1997; Çalikoǧlu et al., 2002; Daga et al., 2003); and cigarette smoke, the primary oxidant, as the chief inducer of it (Rahman et al., 1996; Baginski et al., 2006; Babusyte et al., 2007). Moreover, since blood sample collection is less invasive than lung biopsy or bronchoalveolar lavage collection and also invasive procedures generate significant chances of bronchospasm, we evaluated the antioxidative and anti-inflammatory effects of herbs through blood sample analyses. Our study results are in line with those of previous studies, such as that of Rahman *et al.* (Rahman et al., 1996), showing decreased sulfhydryls, total antioxidant capacity, and increased MDA level in COPD patients. The present study reveals that clinical WS is a strong antioxidant since systemic OS ameliorated the most in WS with the conventional drugs taking patients, in comparison to patients taking only conventional medicines or the patients taking placebo capsules with conventional medicines. Our findings suggest that conventional medicines reduced OS, but the effect was enhanced when WS was added in the regimen. Furthermore, the total antioxidant status and MDA concentration values correlated well with the FEV_1_% predicted values, but other OS parameters did not correlate well with it, thus suggesting a partial contribution of antioxidant nature of WS in improving lung functioning. Previous studies have identified the OS targets and its probable mechanism of damaging the lungs. OS can induce senescence pathways by telomere shortening; inactivation of antiproteases; elevated mucus secretion; and altered histone acetylation, leading to corticosteroid resistance (Laniado-Laborín, 2009; Dove et al., 2015), increase in NF-κB acetylation, and MMP-9 upregulation (Nakamaru et al., 2009). It can also activate NF-κB and transforming growth factor (TGF)-*β* pathways (Daga et al., 2003; Gorowiec et al., 2012). Thus, it is involved in airway fibrosis. Moreover, by upregulating MMP-9 and inactivating *α*1-antitrypsin, it decreases the elasticity of lungs, thus leading to emphysema, which is an irreversible condition (Taggart et al., 2000). This suggests that the pathways are likely to be effected by WS antioxidants and thus have improved lung functioning. Some of these pathways are reversible, and others can be inhibited only at the initial stage and not later. Withaferin A, withanoside V, and withanolide B are some of the known antioxidant molecules for WS.

WS showed a significant decrease in systemic inflammation. In the WS group patients, neutrophil count decreased after treatment. But such improvement was not visible in the control group. A previous *in vivo* study of a mice asthma model supports our finding, where WS showed a significant decrease in the neutrophil count. Moreover, G Cox (Cox, 1995) revealed that corticosteroids inhibit neutrophil apoptosis and increases their survival. This explains the reason for no significant decrease in the neutrophil count in patients taking only conventional medicine. Furthermore, this also indicates that WS is able to overcome the inhibitory effect of corticosteroids. Nitric oxide level was found to be in normal range, but it was at the higher side of it. It is well-known that nitric oxide is anti-inflammatory when in the normal range and becomes proinflammatory at higher concentration. In the WS group, post intervention, its value increased, and it was still in the normal concentration range, while this was not the case in the other two groups. This could be a reflection of the increased anti-inflammatory activity in patients by WS intake. The ELISA results showed that neither conventional drugs nor WS was effective in decreasing the increased level of serum TNF-*α* in COPD patients in 12 weeks of intervention. However, conventional drugs were effective in decreasing the serum IL-6 level significantly, and WS further decreased its level in GOLD 2 category COPD patients when given as add-on to conventional medicines. This suggests that WS is effective in less severe patients for decreasing the systemic inflammatory marker, IL-6. It might show significant results for more severe patients in longer duration of intervention. Moreover, our results are in line with those of previous studies showing that WS can decrease serum IL-6 level *in-vivo* (Minhas et al., 2012).

Myeloperoxidase (MPO), the key enzyme of neutrophils and macrophages, possesses very strong oxidative and inflammatory properties. Primarily, it produces hypochloric acid, a very powerful oxidant. MPO plays a very crucial role in the progression of inflammatory conditions including COPD, such as protein and lipid oxidation (Hazen et al., 1999); NF-κB activation (Van der Veen et al., 2009); release of RNS and inflammatory cytokines, namely, TNF-*α*, IL-6, and IL-8; and neutrophil activation in autocrine manner, ultimately resulting in increased neutrophil infiltration, and it is also involved in advanced glycation end product (AGE) formation, leading to proinflammatory and profibrotic mediator release (Anderson et al., 1999; Reynolds et al., 2008). Earlier, certain MPO inhibitors have been examined *in vitro* and *in vivo* in the COPD models, showing very promising results. It inhibited further progression of smoking-induced airway remodeling (Churg et al., 2012). This indicates a crucial role of MPO in emphysema development. Previous studies have shown that neutrophils of smokers have an elevated MPO level (Bridges et al., 1985). Interestingly, a study found an increased MPO activity in smoker’s neutrophils, which had no significant correlation with the neutrophil count in some smokers (Bridges et al., 1985). Thus, it was important to analyze the MPO activity independently of neutrophil count. In our present study, a highly significant decrease in the MPO activity has been observed. But, it had no significant correlation with neutrophil count (data not shown). The presence of another source of MPO, that is, macrophages/monocytes could be the reason for no significant correlation between neutrophil and MPO activity. Since MPO activity has a pivotal and distinct role in COPD development, decrease in its activity would greatly inhibit further disease progression and decrease OS and inflammation. Furthermore, MPO can also oxidize NO to nitrite and nitrite to nitrogen dioxide, a highly reactive radical leading to protein oxidation and thus, decreases the anti-inflammatory molecule, NO (Abu-Soud and Hazen, 2000; Eiserich et al., 2002). This suggests that post-intervention increase in NO concentration in our study could also be due to the inhibition of MPO activity. (Chapman et al., 2010).

COVID-19 patients with severe conditions suffer from cytokine release syndrome (CRS). They have increased levels of several cytokines, including IL-6, IL-8, and TNF-*α* (Mehta et al., 2020; Ruscitti et al., 2020; Shi et al., 2020). In such conditions, WS could be useful since it has shown to decrease the systemic IL-6 level significantly.

*In silico*, withanolides showed strong ACE-2 inhibitory activity, even stronger than the standard drug, hydroxylchloroquine, suggesting strong anti-SARS activity. As already mentioned, COPD patients have shown severe outcomes by COVID-19. The upregulation of ACE-2 in COPD patient lungs is suggested to be one of the reasons for the same. Thus, by inhibiting the ACE-2 receptor, WS can decrease the chances of severe outcomes and viral entry in host cells. In future, *in vitro* and clinical trials can be conducted for its anti-SARS activity. Moreover, strong binding energy of different withanolides for MPO and IL-6 proteins supports a significant decrease in MPO activity and IL-6 level in patients. No adverse effects were observed in the patients.

## Limitations and Future Perspective

Only GOLD2 and GOLD3 COPD patients were selected for the study. This study did not include GOLD 1 and GOLD 4 category COPD patients. This study was limited to 12 weeks of the intervention period. It would be interesting to see WS effects in a broader category of patients, that is, GOLD 1 and GOLD 4. In future, study can be conducted in larger population including GOLD 1 and GOLD 4 category for a longer period of time to see its effect in different grades of severity in a long term.

## Conclusion

In conclusion, this is the first report showing that WS works very effectively as add-on therapy to conventional medicine in controlling COPD symptoms and improving pathophysiology clinically. It significantly improved lung functioning, quality of life, exercise tolerance, and decreased systemic inflammation and OS. Thus, WS is very effective for COPD treatment. Since conventional medicines have numerous adverse side effects, WS can be evaluated in future for the dose reduction of standard drugs. Thus, our novel findings have paved the way for the development of a superior treatment regimen for ameliorating disease pathogenesis.

## Data Availability

The datasets presented in this study can be found in online repositories. The names of the repository/repositories and accession number(s) can be found in the article/Supplementary Material.
