# Supplementary figures and images for: Withania somnifera (L.) Dunal as Add-On Therapy for COPD Patients: A Randomized, Placebo-Controlled, Double-Blind Study
Source: Front Pharmacol. 2022 Jun 16;13:901710. doi: 10.3389/fphar.2022.901710 (PMC9243480; doi:10.3389/fphar.2022.901710)

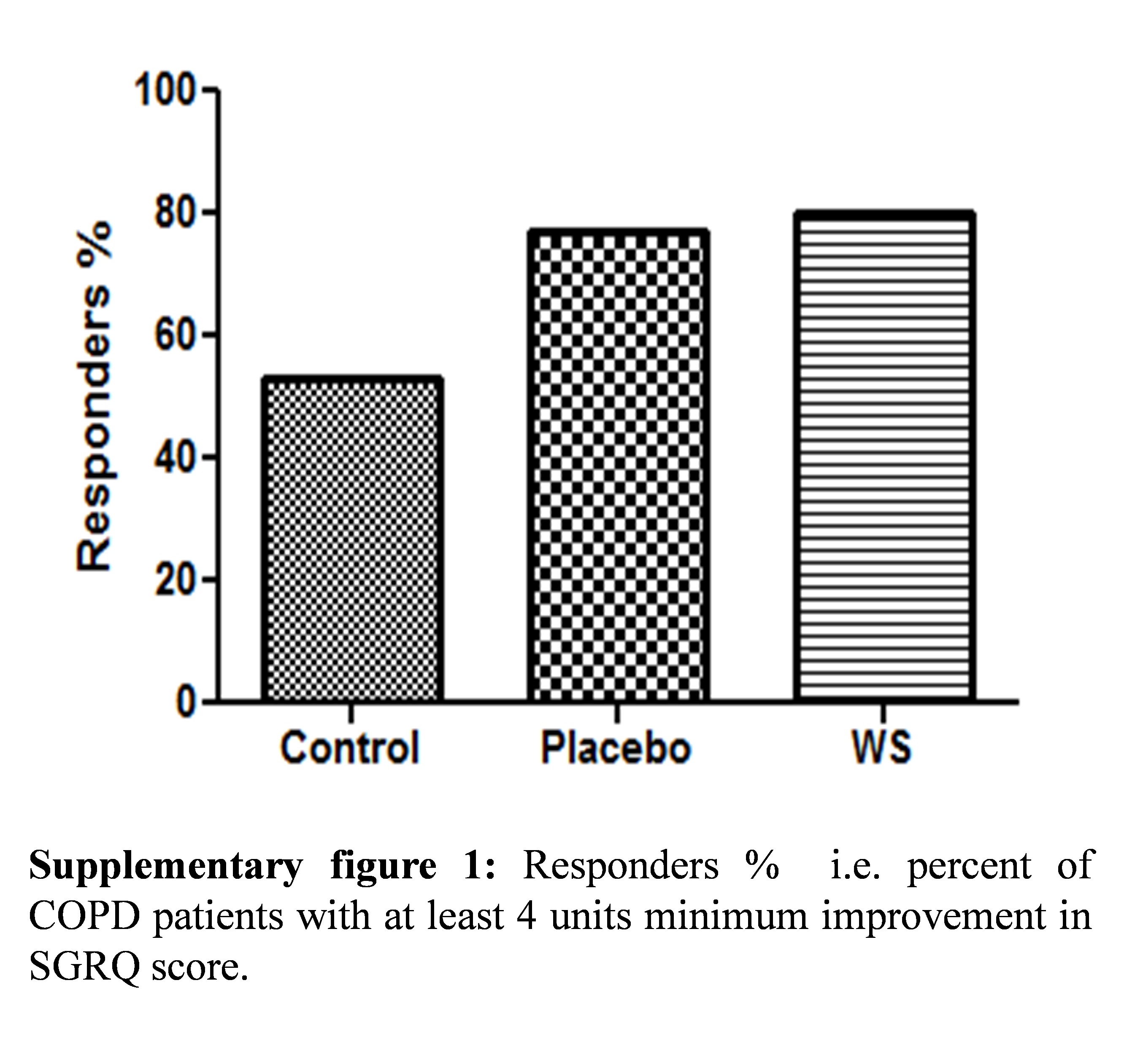

Supplement: Supplementary file 1 [file Image1.jpeg]

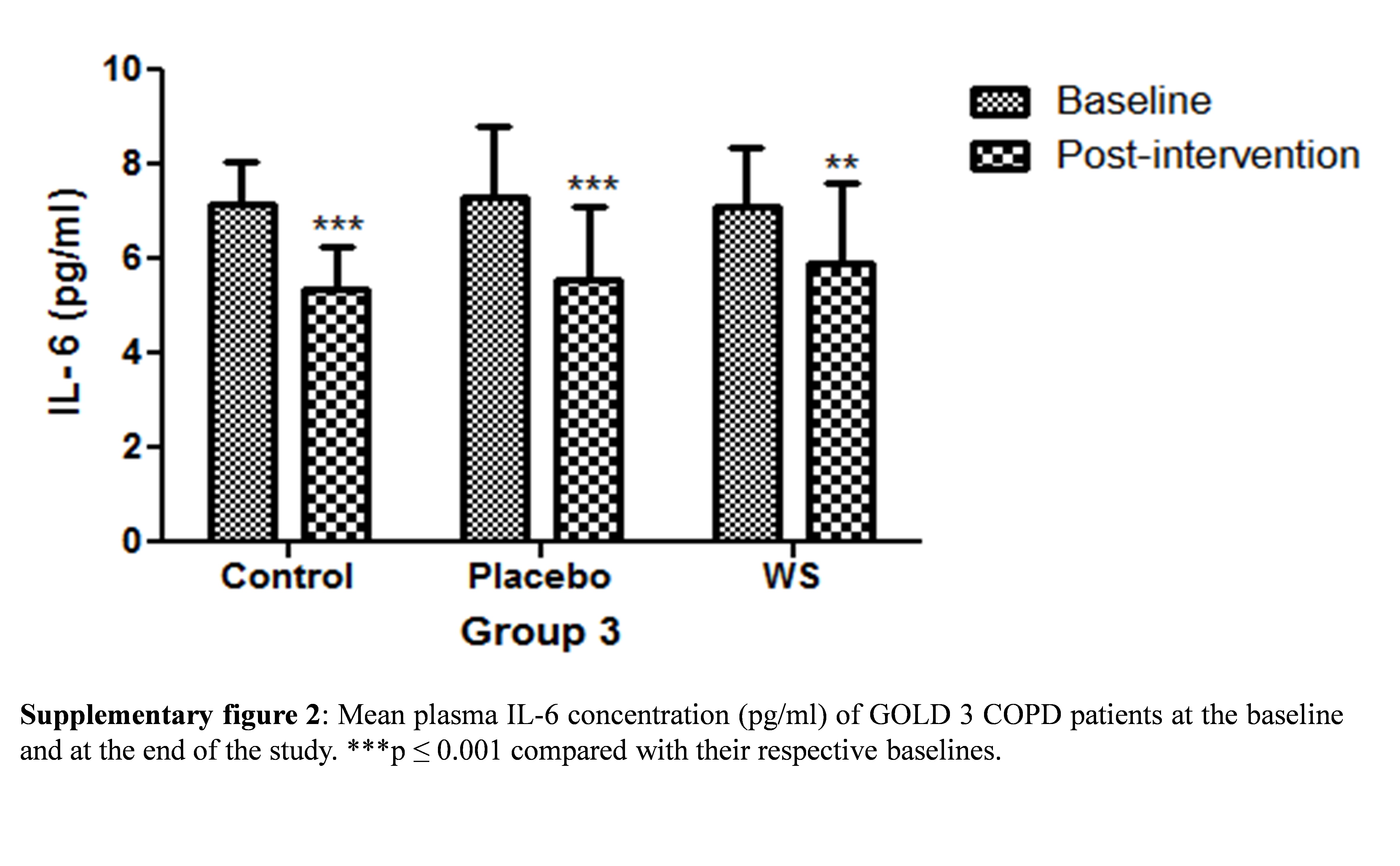

Supplement: Supplementary file 2 [file Image2.jpeg]
